# The Minimally Invasive Posterior Approach: A Novel Technique for Shrapnel Removal in the Knee

**DOI:** 10.1155/cro/8120668

**Published:** 2025-12-15

**Authors:** Mohamed I. Abulsoud, Youssef Alkhier Taha, Mohamed Saleh Hemdan, Akram Mohamed Ali

**Affiliations:** ^1^ Department of Orthopedic Surgery, Faculty of Medicine, Al-Azhar University, Cairo, Egypt, azhar.edu.eg; ^2^ Department of Orthopedic Surgery, King Khalid Hospital, Najran, Saudi Arabia, ksu.edu.sa

**Keywords:** gunshot, knee surgery, minimally invasive posterior approach, periarticular missiles, retained shrapnel

## Abstract

Retained periarticular missiles are usually overlooked, though they could lead to several delayed complications, which include infection, mechanical symptoms, synovitis due to lead particle deposition, lead arthropathy, and systemic lead poisoning. Retrieval of this shrapnel could be open or arthroscopic. The study reports a successful retrieval of retained periarticular shrapnel from the posterior compartment of the knee using the minimally invasive posterior approach. It presents the current literature review regarding this approach and various modifications. In conclusion, the minimally invasive posterior approach is a very good option for retrieving retained shrapnel from the posterior compartment of the knee. It offers good exposure, is relatively safe, and leaves a small scar, but it requires some learning to use efficiently.

## 1. Introduction

Gunshot injuries of the joints are associated with high morbidity compared with other gunshot wounds [1]; periarticular gunshot injuries were defined as any wound, or radio‐opaque blast shrapnel that could be found within 5 cm of a joint [2]; the knee is the most involved area in civilian gunshot or blast injuries [3, 4].

Retained missiles are usually well tolerated. However, periarticular retained shrapnel is associated with peculiar, delayed complications, which include infection, mechanical symptoms, synovitis due to lead particle deposition, lead arthropathy, and plumbism, which is systemic lead poisoning from lead absorption through the synovial fluid [5, 6].

Retrieval of intra‐articular shrapnel could be open or arthroscopic; arthroscopic surgery is more popular to use. It is a relatively safe and reliable option for bullet removal [7]; in the difficulties in performing magnetic resonance imaging, arthroscopy could help in the diagnosis of missed intra‐articular injuries which were not diagnosed by conventional radiographs [8]; however, arthroscopic retrieval could lead to serious complications such as compartment syndrome [9], besides other complications of knee arthroscopy like infection, thrombo‐embolism, instrument breakage, and increased surgery time [10].

This case report is aimed at presenting a case of retained shrapnel in the posterior compartment of the knee, which has been successfully retrieved by a minimally invasive posterior approach to the knee and present the current literature review as regards this approach and various modifications.

## 2. Case Report

A male patient, 32 years old, presented with retained shrapnel in the posterior compartment of the right knee after a gunshot injury 6 months earlier. On examination, there was an inlet wound in the posterior aspect of the right thigh; the main missile had been removed in another center. Knee range of motion was full, and the neurovascular examination had no abnormalities. Plain X‐ray and CT showed the presence of about 8‐mm shrapnel in the posterior aspect of the knee (Figure 1). MRI could not be performed due to the policy of the hospital regarding metallic missiles. Laboratory investigations, including serum lead, were normal.

Figure 1(a, b) Plain X‐ray anteroposterior and lateral views show the retained shrapnel in the posterior compartment of the right knee. (c, d) CT scans in axial and sagittal reformat showing the accurate position of the shrapnel, measuring the largest diameter found to be 8 mm.

**Figure:**
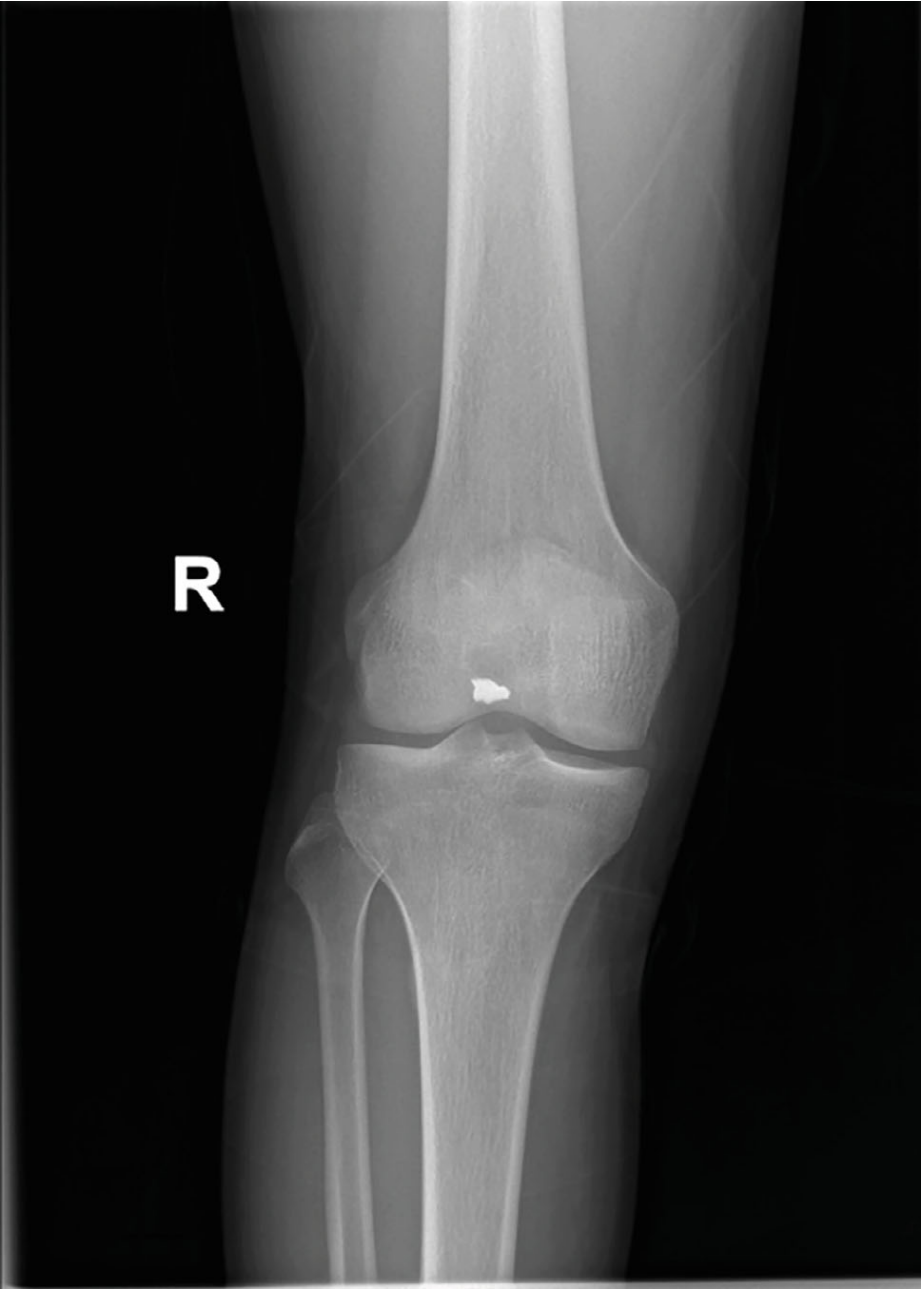
(a)

**Figure:**
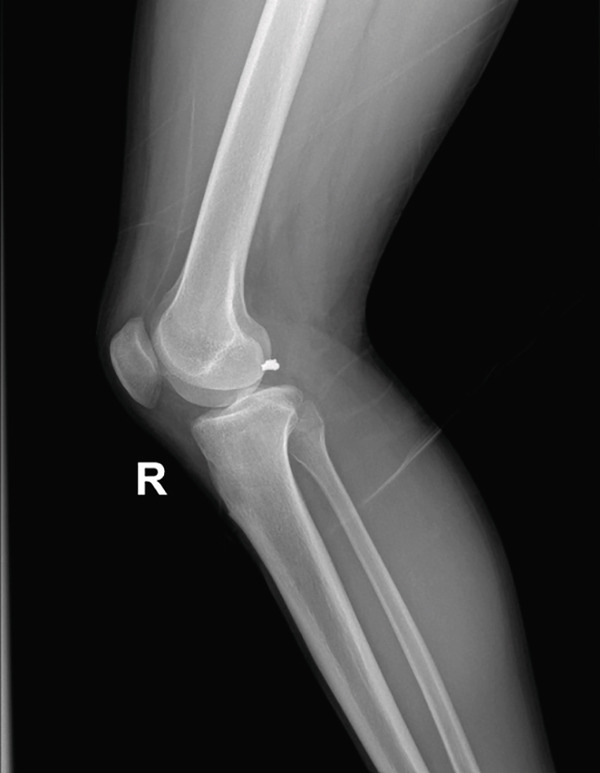
(b)

**Figure:**
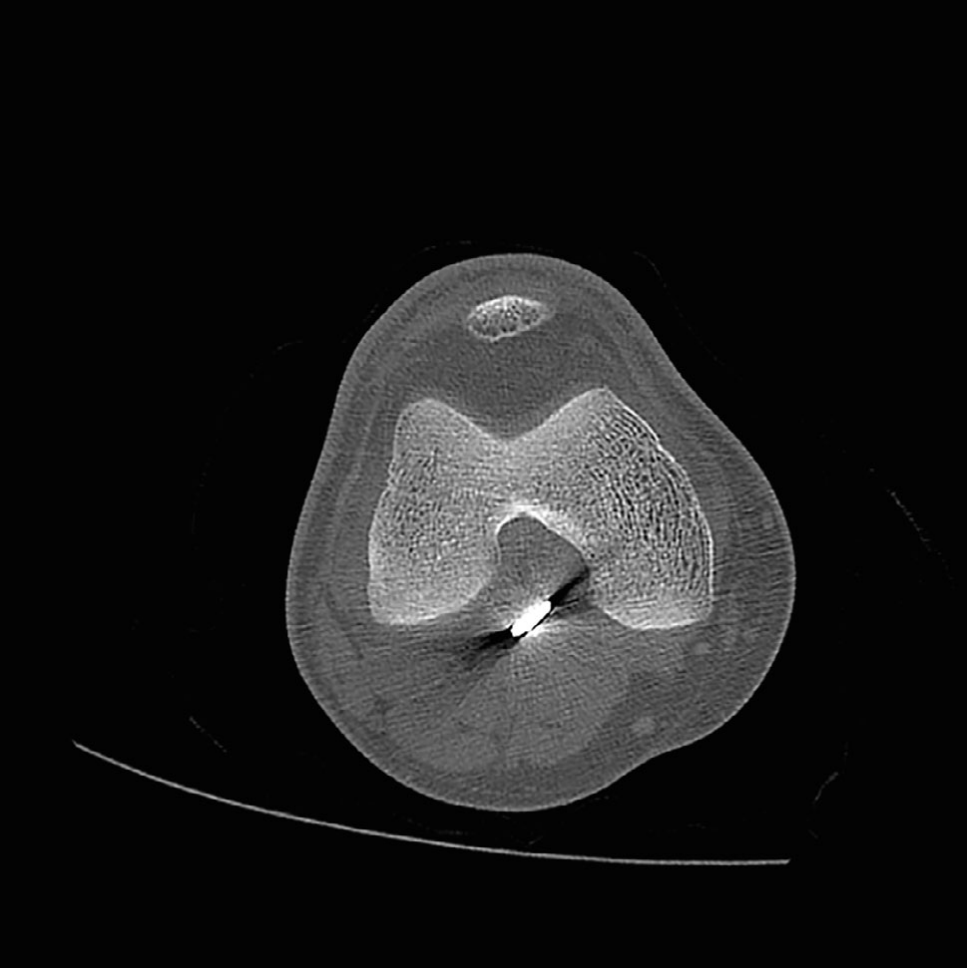
(c)

**Figure:**
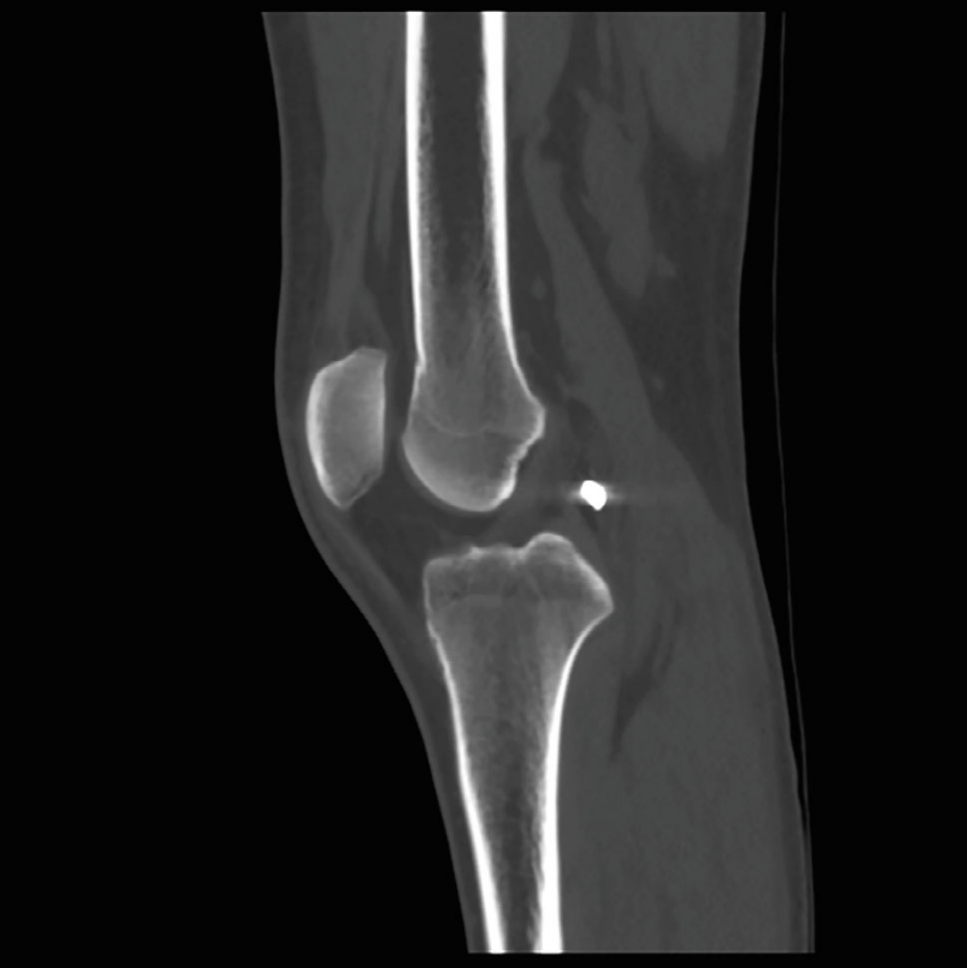
(d)

Surgery has been offered to the patient as an open procedure through a small incision; the patient has been counselled about the possible complications, and written consent has been secured.

Surgery has been performed under spinal anesthesia, in the prone position, with the use of a tourniquet, after adequate sterilization and draping. A 3–4 cm horizontal incision has been made on the posteromedial aspect of the right knee parallel to the posterior crease. Dissection of subcutaneous tissues in line with the skin incision and opening of the fascia has been done. An interval has been made between the medial head of the gastrocnemius muscle and the semitendinosus tendon. With the help of knee flexion, the medial head of the gastrocnemius has been retracted laterally, protecting the neurovascular bundle in the popliteal fossa. The posterior capsule of the knee at this time was identified (Figure 1). The location of the shrapnel has been identified using fluoroscopy. The capsule was opened at this site, and the shrapnel has been retrieved using a curved hemostat. Fluoroscopy was used for confirmation of the complete removal of the shrapnel (Figure 2).

Figure 2(a) The position of the patient is in the prone position, and the dashed lines show the proposed surgical incision; P, proximal; M, medial. (b) Skin incision and subcutaneous tissues; retractors were applied after opening the fascia to show the medial head of the gastrocnemius muscle belly (white Astrex). (c) Fluoroscopic image in anteroposterior view shows the retained shrapnel, and a hemostat is used to determine the location of the capsular incision. (d) Image after retrieval of the shrapnel, a ruler shows the length of the skin incision (about 3 cm).

**Figure:**
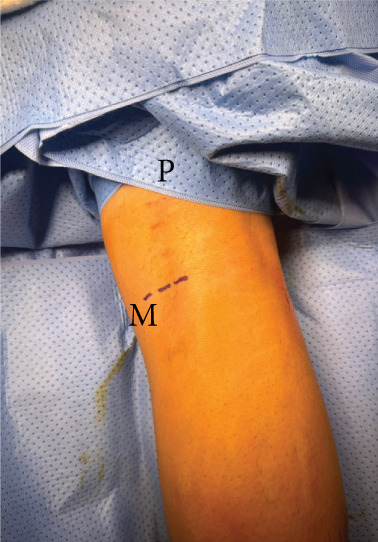
(a)

**Figure:**
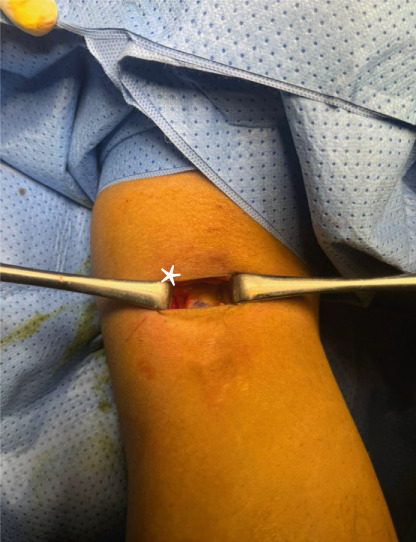
(b)

**Figure:**
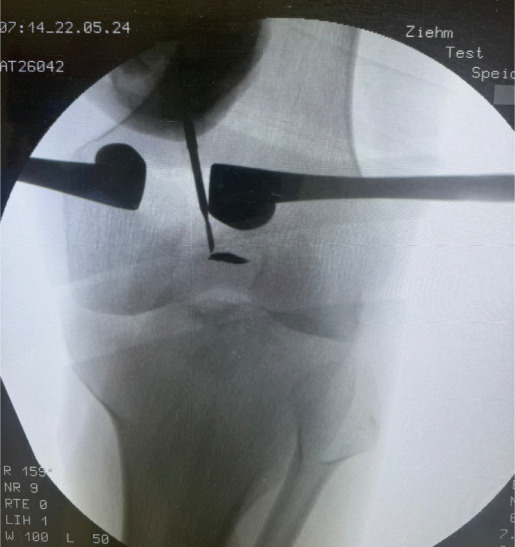
(c)

**Figure:**
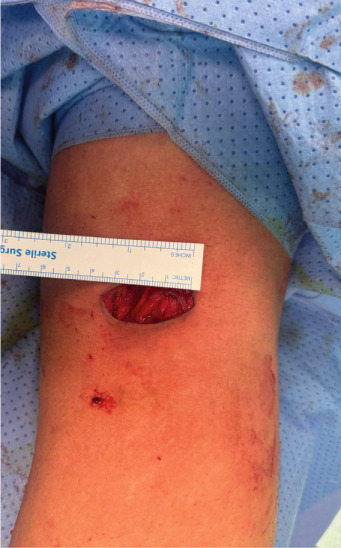
(d)

The tourniquet has been deflated; there was no active bleeding. Wound lavage and skin closure with 3/0 Prolene sutures have been performed. The wound has been cleansed and covered with a sterile dressing.

Postoperatively, an oral antibiotic was used for 72 h, and an anticoagulant, Enoxaparin 0.4 IU, was prescribed for 14 days. With analgesia on demand, the patient was allowed to bear weight as tolerated immediately postoperatively, partially, and weight‐bearing increased as the patient tolerated it.

Stitches were removed at 14 days postoperatively. There were no signs of infection, and the neurovascular examination was intact, with some limitations in knee flexion.

Then, 6 weeks postoperatively, the patient regained his full range of motion, had no active complaints, and was allowed to return to his daily living activities (Figure 3).

Figure 3Functional outcome of the patient 6 weeks postoperatively. (a) Full knee flexion. (b) Full extension. (c) Squatting without restrictions.

**Figure:**
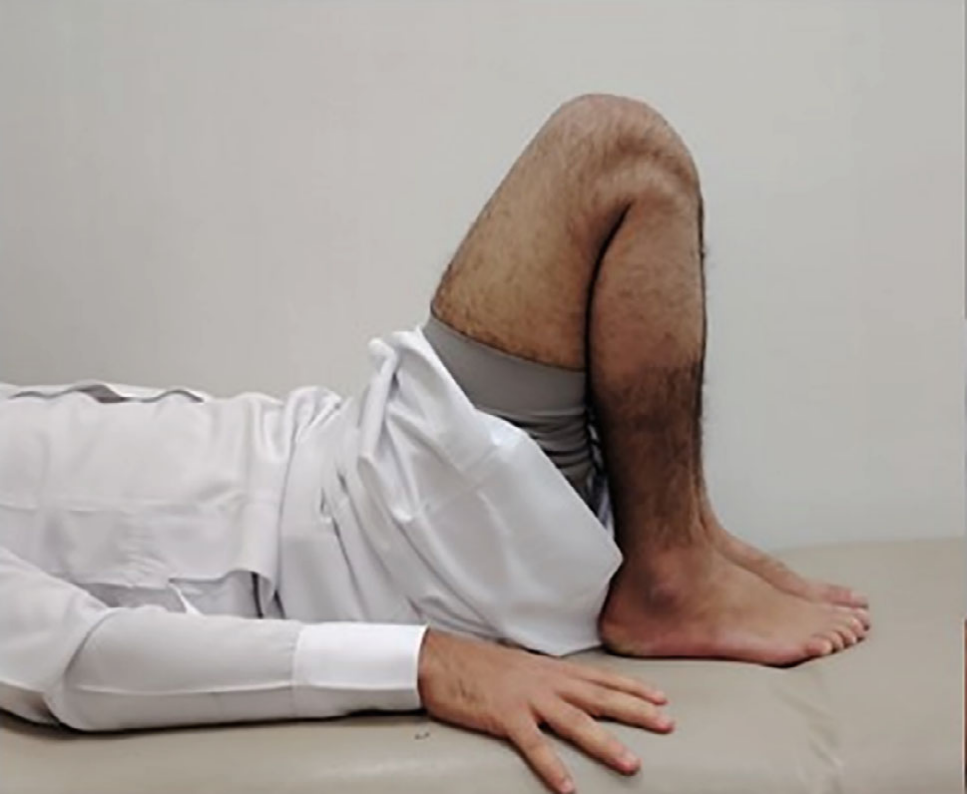
(a)

**Figure:**
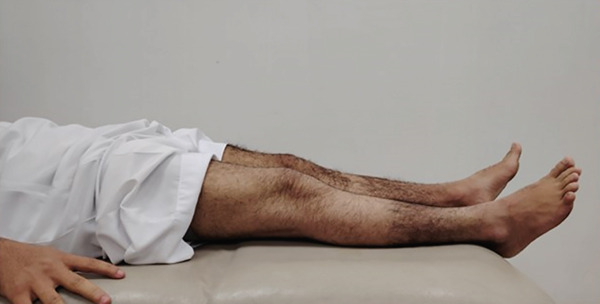
(b)

**Figure:**
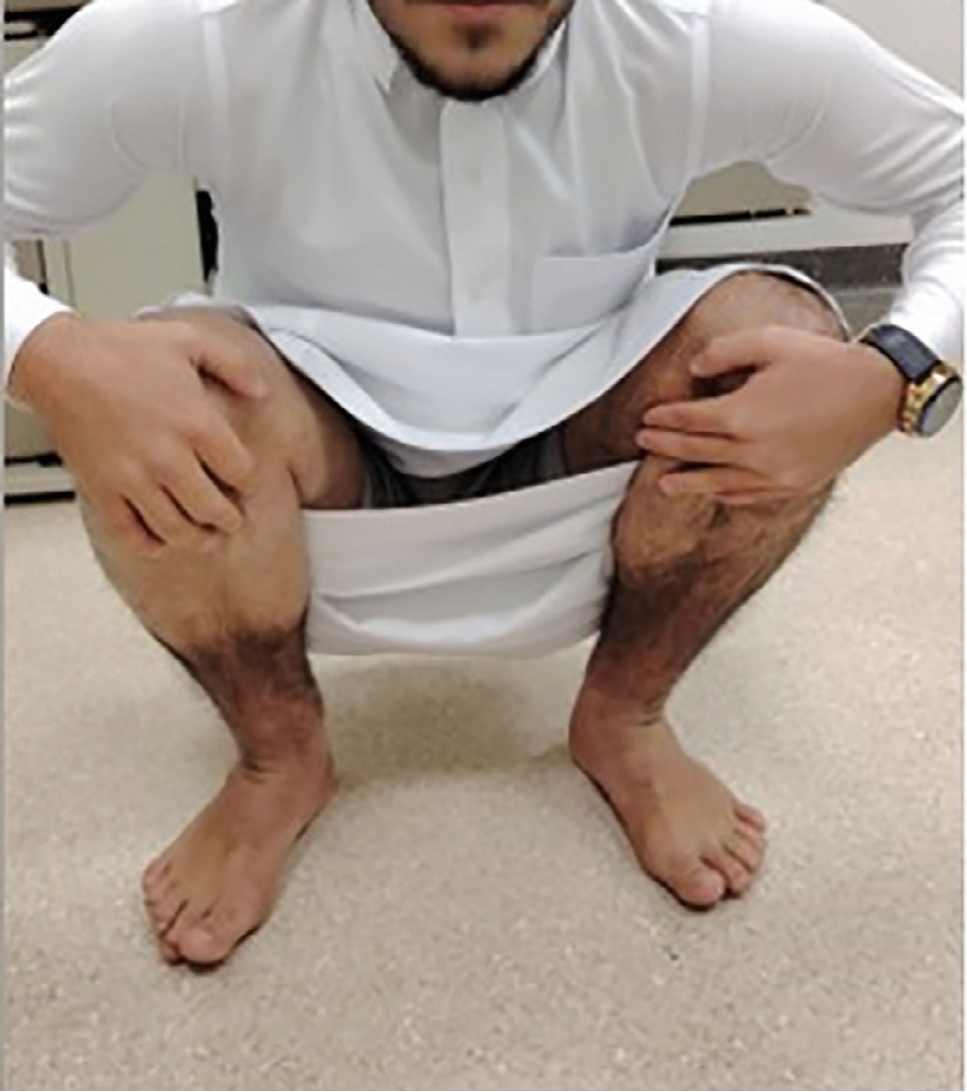
(c)

## 3. Discussion

Retrieval of a retained small shrapnel from the knee is challenging, especially when it involves the posterior compartment of the knee. Both arthroscopic and standard open techniques have advantages and disadvantages [11].

The minimally invasive posterior approach of the knee has been gaining popularity in the last few years, especially in the management of posterior cruciate ligament avulsion fractures. The same technique used in this case report has been used by Zhao et al. [12] by using a small horizontal incision and utilizing the space between the medial head of the gastrocnemius muscle belly and semitendinosus tendon; the same technique also has been described by Guo et al. [13] for addressing the same fracture.

The minimally invasive posterior approach for the management of posterior cruciate ligament avulsion has several modifications in the literature. Singer and Halawa [14] utilized a vertical central skin incision and split the medial head of the gastrocnemius muscle belly into the medial half, which is retracted medially, and the lateral half, which is retracted laterally to protect the neurovascular bundle. While Gavaskar et al. [15] used a horizontal skin incision with fluoroscopic‐assisted references and then utilized the space between the two heads of gastrocnemius to approach the posterior capsule, the neurovascular structures were protected by the self‐retaining lumbar spine microdiscectomy retractor. Zhang et al. [16] used a small, oblique posteromedial skin incision, utilizing the same interval used in this case report, depending on the medial head of gastrocnemius to protect the neurovascular bundle. While Sabat et al. [17] used an inverted L‐shaped skin incision and utilized the space between both heads of the gastrocnemius muscle, Abdallah and Arafa [18] described the same technique using a quite larger skin incision.

Chen et al. [19] used microendoscopy for visualization, using a 2‐cm central skin incision, using the space between the two heads of the gastrocnemius, direct visualization of the neurovascular bundle, and protecting them with a retractor.

In cases of gunshot injuries by ferromagnetic projectiles, magnetic instruments are appealing options to reduce surgical time and fluoroscopic exposure. It is well established in more sensitive areas like the brain and orbital areas with a high success rate, or in combination with endoscopic surgery in thoracic or abdominal injuries, without significant complications [20–22].

As regards strength and weakness points, the minimally invasive posterior knee approach could be a good option as it carries the advantage of being time‐consuming, has no large scar that needs rehabilitation, allows excellent visualization, and does not have the same complications of arthroscopic surgery, especially in the technically demanding posterior compartment. The need for minimally invasive surgery is also important in the management of multiple shrapnel injuries, especially as definitive surgery stages without adding too much endeavour on the personnel or equipment, as it is an open surgery; obviously, there was no need for an arthroscopy tower. However, such an approach needs special experience in the posterior approaches of the knee and adequate orientation with the relevant anatomy of the neurovascular structures and the methods of protecting them.

## 4. Conclusion

The minimally invasive posterior approach is a very good option for retrieving retained shrapnel from the posterior compartment of the knee. It allows good exposure, is relatively safe, and does not leave a large scar, but it needs some learning curve for efficient use.

**Nomenclature**
PCL: posterior cruciate ligament
CT: computerized tomography
MRI: magnetic resonance image

## Ethics Statement

All procedures performed in our study followed the ethical standards of the institutional research committee and the 1964 Helsinki Declaration and its later amendments or comparable ethical standards. The participants obtained informed consent according to the hospital research ethical committee rules.

## Consent

Written informed consent was obtained from the patient for the publication of this case report.

## Conflicts of Interest

The authors declare no conflicts of interest.

## Author Contributions

Mohamed I. Abulsoud: conceptualization, surgical technique, and writing the manuscript. Youssef Alkhier Taha: supervision and writing and revising the manuscript. Mohamed Saleh Hemdan: surgical technique and follow‐up. Akram Mohamed Ali: surgical technique and follow‐up.

## Funding

The authors did not receive funding from a third party, institution, organization, or company.

## Data Availability

The datasets used and analyzed during the current study are available from the corresponding author upon request.
